# Perinatal, neonatal, developmental and demographic predictors of intelligence at 4 years of age among low birth weight children: a panel study with a 2-year follow-up

**DOI:** 10.1186/s12887-022-03156-x

**Published:** 2022-02-12

**Authors:** Flóra Kenyhercz, Karolina Kósa, Beáta Erika Nagy

**Affiliations:** 1 Faculty of Medicine, Department of Behavioural Sciences, University of Debrecen, Debrecen, Hungary; 2 Faculty of Medicine, Department of PediatricsPediatric Psychology and Psychosomatic Unit, University of Debrecen, Debrecen, Hungary

**Keywords:** Infant, Low birth weight, Neonatal Prematurity, Intelligence, Growth & Development, Bronchopulmonary dysplasia

## Abstract

**Intoduction:**

Childhood intelligence is an important predictor of later outcomes in life such as socioeconomic status or health. Hence, a deeper understanding of predictors of child intelligence should suggest points of intervention for children facing adversities.

**Objectives:**

The purpose of this study is to examine the predictive value of demographic, perinatal and neonatal variables after birth and developmental characteristics at age 2 for 4-year intelligence as outcome among low birth weight children.

**Methods:**

We designed a panel study with a 2-year follow-up with 114 child-mother pairs. The outcome variable was IQ intelligence quotient at 4 years of age of LBW low birth weight children measured by the Wechsler Primary and Preschool Scales of Intelligence. Potential predictors were maternal education, family wealth, ethnic identity; sex, twin pregnancy, gestational age, birth weight, Apgar scores, maternal smoking during pregnancy; diagnosis of intravetricular haemorrhage, retinopathy of prematurity, bronchopulmonary dysplasia after birth and cognitive, language and motor development at age 2 measured by one composite score of the three Bayley Scales of Infant and Toddler Development aggregated.

**Results:**

Stepwise backward regression was carried out including significant variables from the bivariate analysis. The best model included 4 predictors which accounted for 57% of the variance of the full IQ intelligence at 4-years of age. Maternal higher education was significant positive, below average family wealth and neonatal diagnosis of bronchopulmonary dysplasia were significant negative predictors in the model after birth. 2-year developmental characteristics such as cognitive, motor and language skills were positive predictors of the IQ intelligence at age 4.

**Conclusion:**

Sociodemographic assessment at birth and developmental assessment at two years of age are of crucial importance to recognize children at high risk for delayed cognitive development. High-risk children should be directed to supportive interventions and their development should be regulary monitored.

## Introduction

Cognitive skills operacionalized as childhood intelligence quotient (IQ) comprise one of the most important predictors of later outcomes in life such as socioeconomic status or health [1–3]. Hence, a deeper understanding of predictors of child intelligence should provide points of prevention for children facing adversities.

A large body of original articles across various types of study populations suggests that parental education, family income, and maternal intelligence are dominant predictors of childhood intelligence. Eriksen et al. (2013) [4] found that parental education, maternal IQ and the child’s gender accounted for 24% of the variance in IQ at 5 years of age [4]. Other studies reported similar results: maternal and paternal education together was considered as a strong predictor (*R*^*2*^ = 0.19) of IQ at age 8 [5], while the sociodemographic status of the family at birth explained almost 10% of the variance in IQ at ages between 7 – 11 [6]. Inaddition to parental demographic factors, postnatal factors such as breastfeeding[7] and physical growth of the child during the first year of life [8] were found to be significantly associated with later IQ [7, 8]. Previous studies of children with low birth weight have found associations between birth weight, low intelligence and attention deficits [9, 10]. These unfavourable outcomes may in part be due to premature gestational age, but other neonatal factors such as chronic diseases and social factors including parental intelligence, parental education, and family wealth may also affect the cognitive outcomes of the child. Early developmental milestones have been identified in several studies as important predictors of adult IQ [11]. The impact of these early developmental characteristics vary across studies which may reflect differences between study samples and investigated predictors. Resolution of many of the complex questions about the later consequences of preterm birth and low birth weight requires prospective follow-up studies investigating potential determinant variables. The present study aims to contribute to this resolution.

The primary purpose of the current study is to examine the predictive value of demographic, perinatal and neonatal variables at childbirth and developmental characteristics at age 2 for 4-year intelligence as the outcome among low birth weight (LBW) children.

## Methods

### Ethics approval and consent to participate

The study received permission from the Hungarian Medical Research Council (33176–2/2017/EKU) following the ethical principles of the WMA Declaration of Helsinki. Written informed consent was obtained from all parent(s)/ legal guardians.

### Study design and participants

We designed a panel study with a 2-year follow-up. The sampling frame consisted of children born in the Department of Obstetrics and Gynaecology of the Clinical Center of the University of Debrecen in Hungary between June 2014 – August 2016 with birth weight below 2500 g and no visual impairment due to severe retinopathy of prematurity. Neonates born with visual impairment of prematurity were excluded since diagnostic tools used to measure developmental characteristics and IQ are not suitable for children with visual impairment. Mothers of the invited children made the decision to participate by providing written informed consent. Inclusion was further differentiated by birth weight. All children born below 1500 g without the exclusion criterion were invited to participate, whereas children between 1500–2500 g birthweight were selected to produce a subsample that fit the gender distribution of the very low birth weight (< 1500 g) group. Data of these two groups were analysed together in this paper.

### Sources of data

Data collection was carried out between September 2016 and January 2020. Perinatal and neonatal data were obtained from the final reports of children in the database of the Neonatology Unit of the Department of Obstetrics and Gynaecology. Measurement data at 2 and 4 years of age were collected by the first author, a trained psychologist, by individually assessing children under the supervision of the senior author. All assessments took place at the Pediatric Psychology and Psychosomatic Unit of the Department of Pediatrics of the Clinical Center of the University of Debrecen.

### Timeline of data collection

Three hundred five mother–child pairs with children 2 years of age were recruited in the first period of study (September 2016—August 2018) when the Bayley Scales of Infant and Toddler Development 3^rd^ Edition test [12] was administered. Of those, 114 mothers whose children reached 4 years of age agreed to participate in the second study period – between September 2018 and January 2020 – when the Wechsler Primary and Preschool Scales of Intelligence 4^th^ Edition IQ test [13] was administered.

Four hundred and forty-six children and their mothers conforming to the inclusion criteria were asked between September 2016 and August 2018 to participate in the study when the children were 2 years of age. Of those invited, 305 (68.3%) participated in the first study period when the Bayley Scales of Infant and Toddler Development 3^rd^ Edition test [12] was administered. Of the mother–child pairs assessed in the first study period, 158 children reached 4 years of age between September 2018 and January 2020. All were invited and 114 (72.1%) agreed to participate in the second assessment, when the Wechsler Primary and Preschool Scales of Intelligence 4^th^ Edition IQ test [13] was administered (second study period).

### Outcome variable

The outcome variable was defined as the children’s IQ at 4-years of age measured by the Wechsler Primary and Preschool Scales of Intelligence – 4^th^ edition (WPPSI-IV [13]). The validated Hungarian version of WPPSI-IV was used which measures IQ consistently and accurately attested by its reliability (Chronbach α = 0.86–0.95) calculated during the adaptation process [14]. All 5 primary subtests were completed: Verbal Comprehension (VCI), Fluid Reasoning (FRI), Visual-Spatial ability (VSI), Processing Speed (PSI), and Working Memory (WMI) from which the score of the Full Scale IQ (FSIQ) was calculated. FSIQ reflects the performance in distinct cognitive domains and is considered the most representative indicator of global intellectual functioning. The theoretical mean score of all subtest indices and the FSIQ are normalized (mean: 100; standard deviation: 15 [13]).

### Predictor variables by domains

- **Demographic variables**: Information on maternal education (primary/ secondary/ higher) and subjective family wealth (below average/ average/ above average) were obtained by a questionnaire completed by the mothers at follow-up. Ethnic identity was classified by one external observer (Hungarian/ Roma).
- **Perinatal characteristics:** The child’s sex, twin pregnancy (yes/no), gestational age (months), birth weight (grams) and Apgar scores at 1 and 5 min (0–10) were obtained from the neonatal final reports retrospectively. The Apgar-score is calculated for newborns at 1 and 5 min after birth based on skin color, heart rate, reflex irritability, muscle tone and respiratory effort ranging between 0–10 where a score of eight or above reflects good health [15]. Information on maternal smoking during pregnancy was assessed by a self-report question and included as binary variable (yes/no) in the statistical analysis.
- **Neonatal variables:** Information on any of 3 postnatal illnesses—intravetricular haemorrhage (IVH), retinopathy of prematurity (ROP) and bronchopulmonary dysplasia (BPD)—was obtained from the neonatal final reports as described elsewhere [16].
- **Developmental characteristics at 2 years:** Neurodevelopmental characteristics of the children were measured by the Bayley Scales of Infant and Toddler Development, 3^rd^ Edition (BSID-III [12]). The test measures the infants’s abilities by 5 subscales and provides objective information on cognitive, expressive and receptive language as well as fine and gross motor development. Raw scores of each subscale are normalized to a mean of 10 and standard deviation of 3. Raw scores can be transformed into three composite scores (Q values), which are normalized to a mean of 100 and standard deviation of 15 and refer to the cognitive, language and motor performance. Composite scores between 85 70 and 70 85 (and raw scores between 4 and 7) with -2 SD to -1SD reflect mild delay, while composite scores below 70 (and raw scores below 4) and more than -2 SD identify children with severe delay [12, 17]. For statistical analysis, an aggregated value was calculated from the mean of the 3 composite scores (∑Cog-Lang-Mot).

### Statistical analysis

Statistical analyses were carried out with IBM SPSS Statistics v23 and Stata/IC 16.1. Univariate analysis of variance was used for calculating the power of the sample and probability of type II error. Pearson correlation was used to test bivariate association with all variables. Point biserial correlation was also carried out to account for binary variables (coded as exposed – 1 or non-exposed – 0) that yielded the same results as Pearson. Second, linear regression analysis and stepwise backward regression were conducted with variables of the four predictor domains (demographic, perinatal, neonatal and developmental characteristics) for the outcome variable. Subscales of the BSID-III were tested separately, and also as aggregate measure. Significance level was set at alpha = 0.05. The models were evaluated by explained variance (R^2^), measure of residuals (root mean square error), and measure of the models’ fit (Akaike’s information criterion). Third, predictors of the best fitting model were identified by stepwise backward regression analysis, setting alpha for the removal of predictors at 0.05.

### Patient and public Involvement

The research question addressed patients’ priorities and interests since low birth weight with its consequences has been an important public health issue in Hungary. Hungary had the sixth highest rate of low birth weight among OECD countries in 2018. Participants were involved neither in study design nor in recruitment. Diagnostic tests at two years of age are performed as part of the two-year status examination of premature infants in outpatient care offered to all LBW children. However, IQ assessment at 4 years of age is not part of the routine assessment of LBW children, this was offered only to participants in the study the results of which were fed back to parents of participating children. Results of the children’s assessments at 2 and 4 years of age were discussed with parents. In cases of mild developmental delay, parents were shown developmental exercises to be performed with the child at home. In cases of severe developmental delay, parents were referred to specialist care for further diagnostic tests and intervention. The assessments performed in the study served as important timepoints for psychological and developmental screening of children at risk and their families. Certain results have already been presented to community health workers in touch with families at high risk of LBW birth. Findings will be disseminated to the professional and lay public in the form of conference presentations and health education materials.

## Results

### Characteristics of the sample

Four hundred and forty-six children and their mothers were invited to participate in the study when the children were 2 years of age. Of those, 305 (68.3%) mother–child pairs agreed to participate in the first study period. Of those, 158 children reached 4 years of age in the second study period, all of whom were invited and 114 (72.1%) agreed to participate. The most common reason for non-participation in the first phase and drop-out in the second phase of the study were relocation to a distant part of the country and not willing to travel back.

Univariate analysis of variance was used for calculating the power of the sample and probability of type II error. The observed power was 96% with *p*-value < 0.001; Beta value was 3%. Of all children included in the study, 114 participated in both assessments at 2 and also at 4 years of age (50 boys, 44%). The children’s birth weight ranged between 450 and 2480 g (M = 1310.08 ± 545.72 g). The mean gestational age (M = 30.00 ± 3.85 months; 22–37 months) reflected the prematurity of the children. Apgar-scores were low at 1 min (M = 6.97 ± 1.74), however, the 5-min values (M = 8.34 ± 1.02) were significantly improved (*p* < 0.001). Perinatal, neonatal, developmental and demographic characterestics of the sample are shown in Table 1.

**Table 1:** Characteristics of the sample by predictor domains and their correlations with IQ at 4 years of age

Correlation with IQ at 4 years of age			
**Indicators of the sample** *N* = 114	Descriptive statistics of variables	correlation coefficient (*r*)	significance (*p*)
**Demographic variables of parents**			
Maternal education (primary, %)	14.91	0.492	< 0.001
Family wealth (below average, %)	6.14	0.392	< 0.001
Ethnicity (Roma, %)	11.40	-0.315	0.001
**Perinatal variables at birth**			
Birth weight (mean ± SD)	1310.08 (545.72)	0.402	< 0.001
Gestational age (mean ± SD)	30.00 (3.85)	0.295	0.001
Sex (Male, %)	43.85	-0.044	0.639
Twin pregnancy (yes, %)	41.20	0.164	0.081
Apgar at 1 min. (mean ± SD)	6.97 (1.74)	0.245	0.014
Apgar at 5 min. (mean ± SD)	8.24 (1.02)	0.257	0.010
Smoking during pregnancy (yes, %)	20.17	-0.407	< 0.001
**Neonatal variables after birth**			
Retinopathy of prematurity (yes, %)	15.70	-0.335	< 0.001
Intraventricular haemorrhage (yes, %)	12.28	-0.242	0.010
Bronchopulmonary dysplasia (yes, %)	14.91	-0.408	< 0.001
**Developmental characteristics at 2 years of age**			
BSID-III Raw scores			
Cognitive skills (mean ± SD)	7.99 (2.76)	0.623	< 0.001
Receptive comm. (mean ± SD)	8.51 (2.43)	0.592	< 0.001
Expressive comm. (mean ± SD)	7.28 (2.67)	0.342	< 0.001
Fine motor skills (mean ± SD)	8.50 (2.57)	0.621	< 0.001
Gross motor skills (mean ± SD)	7.82 (2.47)	0.495	< 0.001
BSID-III Composite scores			
Cognitive skills (mean ± SD)	90.17 (13.99)	0.613	< 0.001
Language skills (mean ± SD)	88.18 (13.07)	0.535	< 0.001
Motor skills (mean ± SD)	89.07 (13.77)	0.632	< 0.001
Mean performance			
∑Cog.-Lang.-Mot.^a^ (mean ± SD)	89.14 (11.85)	0.683	< 0.001

BSID-Bayley Scales of Infant and Todler Development

^a^The mean of Cognitive composite score, Language composite score, and Motor composite scores

Coding of categorical data: Ethnicity: Roma = 1, Hungarian = 0; Sex: Male = 1, Female = 0; Smoking during pregnancy: Yes = 1, No = 0; Neonatal diagnosis of Retinopathy: Yes = 1, No = 0; Neonatal diagnosis of Bronchopulmonary dysplasia: Yes = 1, No = 0; Neonatal diagnosis of Intraventricular haemorrhage: Yes = 1, No = 0

### Bivariate correlations

The full-scale IQ (FSIQ) performance at 4 years of age showed significant correlation with all but two predictor variables (sex of child, twin pregnancy) as shown in Table 1. The strongest correlations were found with developmental charachteristics at 2 years of age (cognitive: *p* < 0.001, finemotor: *p* < 0.001 and receptive communication skills: *p* < 0.001) and maternal education (*p* < 0.001). Significant association of FSIQ was found with maternal smoking during pregnancy (*p* < 0.001), birth weight (*p* < 0.001), gestational age (*p* = 0.001), neonatal diagnosis of BPD (*p* < 0.001), ROP (*p* < 0.001) and IVH (*p* = 0.010), Apgar-scores (at 1 min.: *p* = 0.014; at 5 min.: *p* = 0.010), and demographic variables such as ethnicity (*p* = 0.001) and family wealth (*p* < 0.001).

### Model selection

Full-scale IQ at 4 years of age was found to be normally distributed so it was appropriate for linear regression modeling. Independent variables significantly correlated with the outcome variable were included in the models as predictors. Maternal education and family wealth were transformed into binary variables (maternal education: higher education/below; family wealth: below average/higher). Neonatal morbities such as BPD, ROP and IVH were included in the model as binary varibles (yes/no). A series of regression analysis were conducted in order to find the best model.

First, linear regression was conducted with all 16 predictor variables that showed bivariate correlation with the outcome. The 5 BSID subscales (raw scores) were included separately in this model (Model 1 in Table 2). In this model, only maternal education was predictor for FSIQ. Stepwise backward regression with the same set of variables yielded a slightly stronger model in which five variables (maternal education, family wealth, fine motor, gross motor and receptive communication skills at 2 years of age) remained as significant predictors (Model 2).

**Table 2:** Linear regression modeling

	**Model 1**	**Model 2**	**Model 3**	**Model 4**
	16 variables from bivariate analysis (Bayley 5 subscales at 2 years separately)	16 variables from bivariate analysis (Bayley 5 subscales at 2 years separately)	12 variables from bivariate analysis (Bayley at 2 years aggregated)	**12 variables from bivariate analysis (Bayley at 2 years aggregated)**
**Regression**	linear regression	stepwise backward regression	linear regression	**stepwise backward regression**
**No of significant predictors**	1	5	2	**4**
**Adjusted *R***^***2***^	0.5362	0.5491	0.5474	**0.5701**
**RMSE**	12.292	12.12	12.144	**11.835**
**F**	8.23	25.36	11.08	**34.15**
***p* > *F***	0.0000	0.0000	0.0000	**0.0000**
**AIC**	808.8262	796.4019	803.0702	**790.6543**

*AIC* Akaike information criterion, *BSID* Bayley Scales of Infant and Toddler Development, *RMSE* Root mean square error

Next, linear regression (Model 3) and stepwise backward regression (Model 4) were carried out with the same variables as in Model 1 and 3 except for the Bayley subscales which were included as one aggregate measure calculated as described in methods. As Table 2 shows, the use of the aggregate BSID score resulted in better models than including the subscales separately. Model 3 identified maternal education and the 2-year developmental score as significant predictors of intelligence at 4 years of age. Model 4 was identified as the best model based on the highest proportion of explained variance, lowest measure of residuals (RMSE), and lowest AIC reflecting best fit out of the 4 models.

### Predictors in the best model

Our best model included 4 predictors which accounted for 57% of the variance of the full IQ at 4-years of age (Table 3). Of the four predictors, 3 were assessed at birth (maternal education, subjective family wealth, bronchopulmonary dysplasia [BPD] at birth), and one (the aggregated developmental score based on BSID-III) at age 2. Of the four predictors, two were related to the socio-economic status of the family: maternal education and family wealth. Children of higher educated mothers had an average of 8.8 points higher IQ at age 4 than their peers born to primary or secondary educated mothers. Of the socio-demographic factors, the strongest association was found between below-average family wealth and the children’s IQ at 4 years. Preemies of families with below-average wealth scored on average 11.9 points lower in terms of cognitive skills at age 4. Bronchopulmonary dysplasia after birth predicted an average of 8.4 points lower IQ performance at 4 years of age.

**Table 3:** Predictors in Model 4

**Predictors of 4-year-old FSIQ**		Beta	Std. Error	Standardised Beta	t	sig	95% Conf. Interval	
	(Constant)	24.870	10.163		2.447	0.016	4.695	45.043
**At birth**	**Neonatal diagnosis of Bronchopulmonary dysplasia (BPD)**	-8.458	3.560	-0.167	-2.376	0.019	-15.523	-1.392
	**Higher level of maternal education**	8.806	2.515	0.245	3.501	0.001	3.813	13.798
	**Below average family wealth**	-11.985	5.958	-0.145	-2.012	0.047	-23.811	-0.158
**At 2 years of age**	**Developmental characteristics: mean performance based on the BSID-III cognitive, language and motor composite scores (∑Cog-Lang-Mot)**	0.778	0.114	0.508	6.841	< 0.001	0.552	1.003

Coding of categorical data: Neonatal diagnosis of Bronchopulmonary dysplasia (BPD): Yes = 1, No = 0; Higher level of maternal education: Yes = 1, No = 0; Below average family wealth: Yes = 1, No = 0

Out of the predictors in our final model, developmental characteristics at 2 years of age was found to be a strong predictor of later IQ: 1-point increase of the Q-value in two-year-old BSID average performance results in a 0.77-point IQ increase at 4 years of age (Table 3).

## Discussion

Low birthweight children were followed in our birth cohort study in order to identify significant predictors of cognitive performance assessed as IQ at 4 years of age. 57% of the variance in 4-year IQ was explained by two parental variables (maternal education and subjective family wealth), bronchopulmonary dysplasia after birth, and developmental characteristics of the child at 2 years of age. Higher education of the mother was a significant positive, lower family wealth was a significant negative predictor in the final model. Developmental characteristics assessed at 2 years of age such as cognitive, motor and language skills were strong positive predictors of the IQ at age 4.

Strengths of our study are the panel design with two years of follow-up, and the relatively large sample size. The wide range of collected data including parental and familial factors, perinatal data and developmental assessment at 2 years of age allowed multiple regression modeling of the cognitive outcome at 4 years of age. One limitation of our study is the limited range of parental information. We had neither information on relevant maternal conditions such as nutritional status or illicit drug use, nor on any information about the fathers or the quality of the parental relationship that all potentially have an impact on intrauterine growth. An age-fitted control group could have enabled us to compare the developmental delay of our cohort. However, the development of LBW children should primarily be compared to their own previous status so the lack of control does not hinder the interpretation of our findings.

In contrast with other researchers [4, 18, 19] we did not find birth weight or gestational age to be significant predictors of later IQ among LBW children. In most of these studies no information was provided on parental characteristics which might explain the different results. When evaluating our results it is crucial to consider the fact that our predictors are sequential in the sense of providing a longitudinal perspective. Thus, as opposed to many other studies that looked at socio-demographic and perinatal predictors, our study also included developmental measurements at the end of the second year of life. Our final model shows the direct effect of the selected variables on IQ at 4 years and not any indirect impact mediated by fators during the first two years of life. This might explain why perinatal factors that showed significant correlation with the outcome in bivariate analysis did not remain in the final model. It is notable that birth weight was not a significant predictor in any of our models.

Maternal education and family wealth have been shown to be substantial predictors of later IQ among healthy children (4–6). Our study, similarly to others, identified this relationship not only among healthy children but also in low birth weight children. Regarding maternal education, a college or university degree proved to be a positive factor for the child’s IQ, while below-average subjective family wealth was an important negative predictor for intelligence at age 4. The neonatal diagnosis of bronchopulmonary dysplasia was a risk factor for the outcome in our study, consistent with the finding of other groups [20, 21].

Exploring the underlying causes for delayed intelligence among LBW children with chronic lung disease, it must be noted that preterm birth itself disturbs corticogenesis in the brain [22] with later consequences in the form of behavioral and emotional problems which are compounded if clinical conditions including bronchopulmonary dysplasia are also present in the neonates [23]. Newer research specifically showed that preterm infants with BPD had smaller cerebral white matter volumes and impaired cerebellar development compared to preemies with no BPD [24].

Our findings regarding association between demographic factors of the family and the child’s IQ are in alignment with previous research [4, 5, 25]. Children of highly educated parents tend to have better outcomes along several dimensions of life, such as cognition, education, and health [26, 27]. The underlying reasons for this association are manifold: higher educated parents are more knowledgeable, have better job opportunities, generate higher income, have better living conditions which all contribute to them being able to provide more nurturing environments and better educational opportunities for their children [27, 28] as opposed to families with low income [29]. Parental higher education is also linked to greater knowledge about parenting behaviours, educational and health-promoting factors as well [30]. Our results confirm previous findings regarding the significant positive association between paternal income, family wealth, and the child’s working memory and cognitive proficiency [31, 32].

The field of research in early childhood development has seen a great expansion of knowledge in the past 30 years. Increasing attention is being directed to the importance of the early years of childhood, from the Carnegie Report of 1994 in the USA [33] to the seminal publication of the World Health Organization [34], and the establishment in 2006 of the Center on the Developing Child at Harvard University [35]. More and more national and international bodies recognize the vital importance of early childhood, particularly the first three years, in the creation of healthy adults. However, children cannot live and cannot be helped without parents or caretakers. Our study, along with many others, points to the fundamental importance of the quality of the family environment and maternal education as major predictors of the cognitive development of the child. We did not find low birth weight to be the predictor of 4-year intelligence which raises the possibility that LBW may not be a mediator of later intelligence of which the socioeconomic status of the family is determinant, just as LBW is not a mediator between higher socioeconomic level of the family and decreased infant mortality [36].

Interactions between biological (e.g. genetics or heredity; in this case BPD due to prematurity as biological vulnerability) and environmental (e.g. micro- and macro-environment; such as parental education or poverty) factors lead to high variability in developmental outcomes. Tucker-Drob, Briley & Harden (2013) [ highlight that “*genetic influences on cognition are maximized by environmental opportunity”* [37]. The task of researchers is to uncover the determinants of child development and identify effective ways to improve developmental processes. However, planning and implementing interventions by which all families can improve the conditions in which they raise their children is a task for policy makers. A recent publication of the World Health Organization provides guidelines for improving early childhood development based on a review of scientific evidence [38]. Interventions teaching responsive caregiving to parents improve early child outcomes, among others cognitive development measured by the same tool which we used (Bayley Scale of Infant Development), and language and motor development. In Hungary, early childhood development has been helped in a number of ways. An extensive system of maternal benefits enables mothers to care for their children while on paid leave up to the child’s 3 years of age. Kindergarten education became mandatory from age 3 in 2015 (before that, mandatory age was 5 years). Improvement of parental caregiving is one of the goals of a nationwide government-funded initiative launched in 2004 which was modelled on the Sure Start Programme of the UK. However, this Programme has less professionals than it would need especially in geographical areas in which the proportion of vulnerable populations in low socio-economic strata tend to be high [39].

One of our findings is neonatal bronchopulmonary dysplasia as a significant predictor of the developmental and cognitive performance of the child in later years. The incidence of BPD increases with decreasing gestational age and decreasing birth weight. BPD is the most prevalent complication of prematury among infants born before 28 weeks of gestational age ranging between 11–50%, and this constitutes the major cause of mortality [40]. BPD presents a medical challenge for which a number of treatment modalities have been tried with limited success [41]. The public health approach to decrease BPD is to increase gestational age and decrease low birth weight as detailed in a policy brief of the World Health Organization [42].

Our findings show that developmental assessment at two years of age is crucially important to recognize delays and intervene to prevent further delays in later cognitive functioning, a risk factor for learning difficulties. Our data help prioritize those children who should be regularly monitored and supported in their cognitive development: those with mothers having less than higher education, those in families with below-average subjective wealth, and those with BPD with our without the previous two risk factors. Improving the skills of caregiving persons and the quality of the caregiving environment for all infants but especially for those of low birth weight by effective interventions not only reduces inequalities and is the humane thing to do but it is also cost-effective from a societal point of view [43].

## Data Availability

Additional data are available from the corresponding author on reasonable request.

## Abbreviations

BPD: Bronchopulmonary Dysplasia
BSID: Bayley Scales of Infant and Toddler Development
ELBW: Extremely Low Birthweight
FSIQ: Full Scale IQ
IQ: Intelligence Quotient
IVH: Intraventricular Haemorrhage
LBW: Low Birthweight
ROP: Retinopathy of Prematurity
VLBW: Very Low Birthweight
WHO: World Health Organization
WPPSI: Wechsler Preschool Primary Scales of Intelligence
